# Anatomical resection improves relapse-free survival in colorectal liver metastases in patients with *KRAS/NRAS/BRAF* mutations or right-sided colon cancer: a retrospective cohort study

**DOI:** 10.1097/JS9.0000000000000562

**Published:** 2023-08-01

**Authors:** Wenju Chang, Yijiao Chen, Shizhao Zhou, Li Ren, Yuqiu Xu, Dexiang Zhu, Wentao Tang, Qinghai Ye, Xiaoying Wang, Jia Fan, Ye Wei, Jianmin Xu

**Affiliations:** a Colorectal Cancer Center; b Department of General Surgery; c Department of Liver Surgery; d Cancer Center, Zhongshan Hospital, Fudan University; e Shanghai Engineering Research Center of Colorectal Cancer Minimally Invasive Technology, Shanghai; f Department of General Surgery, Zhongshan Hospital (Xiamen Branch), Fudan University, Xiamen, People’s Republic of China

**Keywords:** anatomical resection, colorectal liver metastases, *KRAS/NRAS/BRAF*, relapse-free survival, right-sided colorectal cancer

## Abstract

**Background:**

The type of liver resection (anatomical resection, AR or non-anatomical resection, NAR) for colorectal liver metastases (CRLM) is subject to debate. The debate may persist because some prognostic factors, associated with aggressive tumor biological behavior, have been overlooked.

**Objective:**

Our study aimed to investigate the characteristics of patients who would benefit more from anatomical resection for CRLM.

**Methods:**

Seven hundred twenty-nine patients who underwent hepatic resection of CRLM were retrospectively collected from June 2012 to May 2019. Treatment effects between AR and NAR were compared in full subgroup analyses. Tumor relapse-free survival (RFS) was evaluated by a stratified log-rank test and summarized with the use of Kaplan–Meier and Cox proportional hazards methods.

**Results:**

Among 729 patients, 235 (32.2%) underwent AR and 494 (67.8%) underwent NAR. We showed favorable trends in RFS for AR compared with NAR in the patients with *KRAS/NRAS/BRAF* mutation (interaction *P*<0.001) or right-sidedness (interaction *P*<0.05). Patients who underwent AR had a markedly improved RFS compared with NAR in the cohorts of *RAS/NRAS/BRAF* mutation (median RFS 23.2 vs. 11.1 months, *P*<0.001) or right-sidedness (median RFS 31.6 vs. 11.5 months, *P*<0.001); upon the multivariable analyses, AR [gene mutation: hazard ratio (HR)=0.506, 95% CI=0.371–0.690, *P*<0.001; right-sidedness: HR=0.426, 95% CI=0.261–0.695, *P*=0.001) remained prognostic independently. In contrast, patients who underwent AR had a similar RFS compared with those who underwent NAR, in the cohorts of patients with gene wild-type tumors (median RFS 20.5 vs. 21.6 months, *P*=0.333). or left-sidedness (median RFS 15.8 vs. 19.5 months, *P*=0.294).

**Conclusions:**

CRLM patients with gene mutation or right-sidedness can benefit more from AR rather than from NAR.

## Graphical abstract

**Figure:**
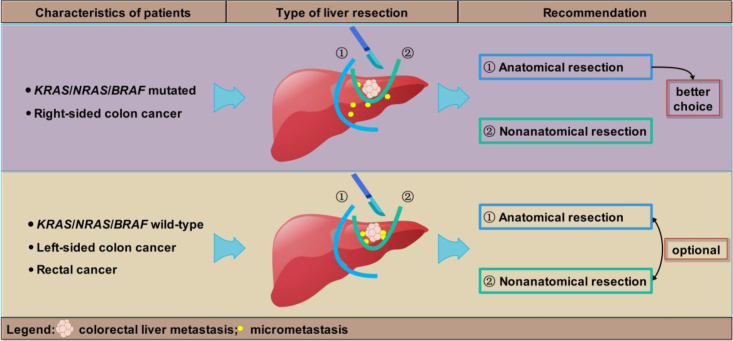

## Introduction

Highlights

- This study is the first to show that anatomical resection (AR) improves relapse-free survival (RFS) in colorectal liver metastases (CRLM) patients with right-sidedness.
- In this study of 729 patients who underwent hepatic resection of CRLM, our finding about CRLM patients with *KRAS/NRAS/BRAF* mutations or right-side colorectal cancer may benefit from the performance of AR.
- Margonis and colleagues reported that AR improved disease-free survival in patients with *KRAS* mutant CRLM. We found that not only *KRAS* but also *NRAS* and *BRAF* mutant CRLM patients benefited from anatomical resection in RFS.

Worldwide, colorectal cancer (CRC) is the third most commonly diagnosed malignancy, with 1.8 million new CRC cases diagnosed annually^1^. Approximately 50% of CRC patients would develop liver metastasis, resulting in a significant mortality rate^2^. Hepatic resection of colorectal liver metastases (CRLM) offers the best chance for cure and long-term survival^2^. Nevertheless, the postoperative relapse rate is still higher than 50% within the first two post-hepatectomy years^3^. Precision surgical strategies could reduce relapse rates and improve survival outcomes^4^.

Based on the segmental anatomy of the liver or not, hepatic resection can be characterized as anatomical resection (AR) or non-anatomical resection (NAR). For CRLM, the type of hepatic resection (AR or NAR) is subject to debate. Contemporary tendencies lean toward NAR^5^. Sarpel *et al.* ^6^ reported that there was no significant difference between AR and NAR for CRLM in terms of recurrence or survival. A systematic review of 12 studies and a meta-analysis of 5207 patients found that NAR had a comparable safety and efficacy profile to AR and did not impair oncologic outcomes^7,8^. In addition, Mise *et al*.^9^ discovered that ‘NAR did not increase recurrence in the liver remnant but more importantly improved 5-year survival in case of recurrence (salvageability)’. NAR, also called as a parenchymal-sparing hepatectomy, can facilitate the preservation of hepatic parenchyma. With increased preservation of hepatic reserve, patients who underwent NAR would have a greater chance of undergoing a second hepatectomy if the disease recurred in their liver residual^5,9^. However, AR was previously recommended above NAR due to superior tumor clearance and enhanced long-term survival^10,11^. A complete hepatic anatomical resection, accompanied by the systematic removal of potentially ‘tumor-bearing’ portal tributaries, could control hematogenous micrometastasis in the liver segments^12–15^. AR should be valued for a complete lesion radical resection.

AR may provide more treatment benefits than NAR for certain patients guided by molecular biology. Recently, Margonis and colleagues^12^ reported that AR improved disease-free survival in *KRAS*-mutated CRLM patients. *KRAS* mutations represent a more aggressive biological behavior of tumors, which increases the likelihood of vascular invasion and hematogenous spread^12^. There are further factors associated with tumor biological behavior in CRC, such as *NRAS* mutation, *BRAF* mutation, and right-sided colon cancer (right-sidedness)^16^. These certain factors may have confounded the analyses of previous studies and concealed the potential oncologic benefits of AR. If these considerations are overlooked, the debate on the type of hepatic resection may continue.

Accordingly, we hypothesized that some tumor biological behavior-related factors influence the potential oncologic benefits of AR. We assessed the relapse rate between CRLM patients undergoing AR and NAR using full subgroup analyses. The characteristics of patients who would benefit more from the performance of AR were screened out and investigated further.

## Methods

### Study population

The study was a retrospective cohort study based on our prospectively collected colorectal cancer database. This work was reported in accordance with the STROCSS criteria^17^ (Supplemental Digital Content 1, http://links.lww.com/JS9/A829). All adult patients who underwent curative-intent liver resection for CRLM between June 2012 and May 2019 at our center and had available data on *KRAS/NRAS/BRAF* status were eligible for inclusion. The following exclusion criteria were implemented: (a) the histologic type of tumor was not called adenocarcinoma; (b) peritoneal metastasis; (c) number of liver metastases >3; (d) simultaneous anatomical and non-anatomical resections; (e) R2 resection; (f) a history of previous hepatectomy; (g) incomplete data. The enrolled patients were separated into two groups: those who underwent an AR and those who underwent a NAR. Patients’ demographic information, primary tumor and liver metastases characteristics, clinical information, and follow-up information were all recorded in the electronic medical database. This study was approved by the Ethics Committee of our center in accordance with the Declaration of Helsinki, and it was registered at ClinicalTrials.gov (Registration Number: NCT05673564).

### Definition of AR and NAR

Based upon the segmental anatomy of the liver according to the Couinaud system, AR is defined as the resection of one or more complete hepatic segments in our study, including bisegmentectomy, right hemihepatectomy, left hemihepatectomy, extended right hemihepatectomy, extended left hemihepatectomy, single segmentectomy, caudate lobectomy, or a combination thereof. NAR, also called wedge resection, is defined as the resection of the tumor with a margin of normal parenchyma regardless of the hepatic anatomy. The selection of the AR or NAR approach was prudently determined by the surgeons of our center during the preoperative evaluation.

### Determination of *KRAS/NRAS/BRAF* mutations

*KRAS/NRAS/BRAF* mutational analysis was performed as previously described^18,19^. Formalin-fixed paraffin-embedded (FFPE) tissue was obtained from the Department of Pathology. An experienced pathologist reviewed each section and indicated the area of the tumor. Macro-dissection was performed using the H&E (hematoxylin and eosin)-stained slides to enrich the number of tumor cells in each sample. The gene mutations were detected using the AmoyDx *KRAS/NRAS/BRAF* Mutations Detection Kit (AmoyDx, Xiamen, China), based on Amplification Refractory Mutation System (ARMS) technology. The ARMS technology is a PCR-based method that is used to detect specific mutations or genetic variations in a DNA sample^20^. The ARMS technology works by designing primers that are specific to the mutated or variant allele of interest. When these designing primers are used in a PCR reaction, they amplify only the DNA fragment containing the mutation or variant, while not amplifying the wild-type or normal allele. All types of mutation loci of *KRAS*, *NRAS*, and *BRAF* to be tested were listed in Supplementary Table S1 (Supplemental Digital Content 2, http://links.lww.com/JS9/A830). The detailed steps of ARMS technology are listed after Supplementary Table S1 (seen in Supplementary materials, Supplemental Digital Content 2, http://links.lww.com/JS9/A830). According to the presence of *KRAS/NRAS/BRAF* mutations, the cohorts were divided into the gene-mutated cohorts and the gene wild-type cohorts.

### Categorization of primary tumor site

Primary CRC tumors were classified into two categories according to the embryonic origin: right-sided CRC, primary tumors originating in the midgut origin (cecum, ascending colon, hepatic flexure, and transverse colon); left-sided CRC, primary tumors originating in the hindgut origin (splenic flexure, descending colon, sigmoid colon, and rectum). In this instance, the rectum is characterized as left-sided CRC from an embryological standpoint. According to the primary tumor site, the cohorts were divided into the right-sided CRC cohorts and the left-sided CRC cohorts.

### Follow-up

Patients were followed up mainly through outpatient clinic visits every 2–3 months for the first 2 years after the operation, every 6 months for the next 3 years, and once per year thereafter. Physical examination, serum CEA and CA19-9 tests, chest computed tomography (CT) scan, and abdominal CT and MRI were performed at each follow-up, as well as colonoscopy once per year after the surgery. The starting point of follow-up was the initial liver resection, and the endpoint of follow-up was the evidence of tumor relapse or death until the deadline of 1 October 2020. The specific site of all relapses was recorded and were classified as intrahepatic (involving the liver) or extrahepatic (extrahepatic relapses without liver involvement).

### Statistical analysis

The difference between groups was assessed by *t*-tests, Mann–Whitney *U*, chi-square (*χ* ^2^), or Fisher’s exact tests when appropriate. The Kaplan–Meier method was applied in relapse-free survival (RFS) and intrahepatic RFS analyses to assess the oncologic outcome. AR and other factors with a *P*<0.10 in univariable analysis were included in the multivariable analysis. The Cox proportional hazards regression model was used to identify independent predictors of prognosis in multivariable analysis. Hazard ratios (HR) and 95% confidence intervals (95% CI) were calculated using the Cox proportional hazards model. Heterogeneity among covariate levels in each subgroup was assessed in the subgroup analyses. For each subgroup, the Kaplan–Meier method was fitted. The interaction tests were performed for all subgroup analyses to assess whether the treatment effect varied according to subgroup (i.e. whether the effectiveness of AR differed in right-sided CRC patients compared to left-sided CRC patients). Two-tailed tests were used, and a *P* value less than 0.05 was considered statistically significant. IBM SPSS software version 24.0 (IBM, Armonk, New Jersey, USA) and R software (https://www.r-project.org) were used for statistical analysis. The subgroup analysis of survival was conducted with the R package “Publish” (Version 2020.11.30). The forest plots were plotted with the R package “forestplot” (Version 1.10). The survival curve was plotted with the R package “survminer” (Version 0.4.8).

## Results

### Patients and cohorts

From June 2010 to May 2019, the CRLM Cohort included 729 patients who had hepatic resection for CRLM. Of these, 235 (32.2%) underwent AR and 494 (67.8%) underwent NAR. The patients were stratified into subgroup cohorts based on the presence of *KRAS/NRAS/BRAF* mutations and the primary tumor site (Supplementary Fig. S1, Supplemental Digital Content 2, http://links.lww.com/JS9/A830): (a) gene-mutated CRLM Cohort (*N*=343, Supplementary Table S4, Supplemental Digital Content 2, http://links.lww.com/JS9/A830); (b) gene wild-type CRLM Cohort (*N*=386, Supplementary Table S5, Supplemental Digital Content 2, http://links.lww.com/JS9/A830); (c) right-sided CRC CRLM Cohort (*N*=145, Supplementary Table S6, Supplemental Digital Content 2, http://links.lww.com/JS9/A830); (d) left-sided CRC CRLM Cohort (*N*=584, Supplementary Table S7, Supplemental Digital Content 2, http://links.lww.com/JS9/A830). The CRLM patients in these subgroup cohorts were categorized as either AR or NAR. There was no significant difference between the AR and NAR groups in terms of patient demographics or primary tumor characteristics. Regarding CRLM characteristics, the patients undergoing AR were more likely to exhibit a larger number of CRLM, larger CRLM size, and unilateral CRLM. In practical practice, the surgeon’s choice between AR and NAR was typically determined by the three aforementioned CRLM characteristics. On the basis of ARMS technology, *KRAS*/*NRAS*/*BRAF* mutational analyses were performed for all patients, and the frequencies of exon mutations were recorded in Supplementary Table S2 (Supplemental Digital Content 2, http://links.lww.com/JS9/A830). In the total population, 42.0% of patients have *KRAS* mutation, 3.2% have *NRAS* mutation, and 2.3% have *BRAF* mutation. The patients undergoing AR had a lower percentage of *KRAS/NRAS/BRAF* mutated tumors (Table 1). The information about the quality of liver resection, short-term outcomes, and chemotherapy were summarized in Supplementary Table S3 (Supplemental Digital Content 2, http://links.lww.com/JS9/A830).

**Table 1:** Clinical characteristics of enrolled patients.

	CRLM Cohort			
Characteristics	Total (*N*=729)	AR (*N*=235)	NAR (*N*=494)	*P*
Patient characteristics, *n* (%)				
Age >60 years	424 (58.2)	143 (60.9)	281 (56.9)	0.310
Female	224 (30.7)	75 (31.9)	149 (30.2)	0.632
Primary tumor characteristics, *n* (%)				
Right-sided CRC	145 (19.9)	48 (20.4)	97 (19.6)	0.803
Left-sided CRC	584 (80.1)	187 (79.6)	397 (80.4)	
T stage: T1–T2	67 (9.2)	26 (11.1)	41 (8.3)	0.284
T stage: T3–T4	634 (87.0)	205 (87.2)	429 (86.8)	
T stage: unknown	28 (3.8)	4 (1.7)	24 (4.9)	
N stage: node-negative	218 (29.9)	79 (33.6)	139 (28.1)	0.131
N stage: node-positive	511 (70.1)	156 (66.4)	355 (71.9)	
Preoperative factors, *n* (%)				
Preoperative chemotherapy	240 (32.9)	74 (31.5)	166 (33.6)	0.570
Preoperative CEA >200 ng/ml	57 (7.8)	19 (8.1)	38 (7.7)	0.854
Preoperative CA19-9 >200 U/ml	103 (14.1)	37 (15.7)	66 (13.4)	0.388
*KRAS/NRAS/BRAF* mutated, *n* (%)	343 (47.1)	96 (40.9)	247 (50.0)	0.021
*KRAS* mutated	306 (42.0)	88 (37.4)	218 (44.1)	0.087
*NRAS* mutated	23 (3.2)	4 (1.7%)	19 (3.8)	0.122
*BRAF* mutated	17 (2.3)	4 (1.7)	13 (2.6)	0.437
CRLM characteristics, *n* (%)				
Number of CRLM				
1	435 (59.7)	159 (67.7)	276 (55.9)	0.001
2	191 (26.2)	57 (24.3)	134 (27.1)	
3	103 (14.1)	19 (8.1)	84 (17.0)	
Size of largest CRLM≥5 cm	194 (26.6)	98 (41.7)	96 (19.4)	0.000
Bilateral CRLM^a^	161 (22.1)	31 (13.2)	130 (26.3)	0.000
Synchronous CRLM	523 (71.7)	173 (73.6)	350 (70.9)	0.438
Extrahepatic disease, *n* (%)	72 (9.9)	22 (9.4)	50 (10.1)	0.748
Fong score, *n* (%)				
Low-risk	199 (27.3)	66 (28.1)	133 (26.9)	0.103
Medium-risk	460 (63.1)	139 (59.1)	321 (65.0)	
High-risk	70 (9.6)	30 (12.8)	40 (8.1)	
Surgical procedure, *n* (%)				
Resection only	695 (95.3)	231 (98.3)	464 (93.9)	0.009
Resection plus ablation	34 (4.7)	4 (1.7)	30 (6.1)	

a If the CRLM is located on the boundary line between the left lobe and the right lobe of liver, it is considered as bilateral metastasis.

AR, anatomical resection; CEA, carcinoembryonic antigen; CRLM, colorectal liver metastasis; NAR, non-anatomical resection.

### RFS and intrahepatic RFS in the total CRLM Cohorts

**Figure 1:**
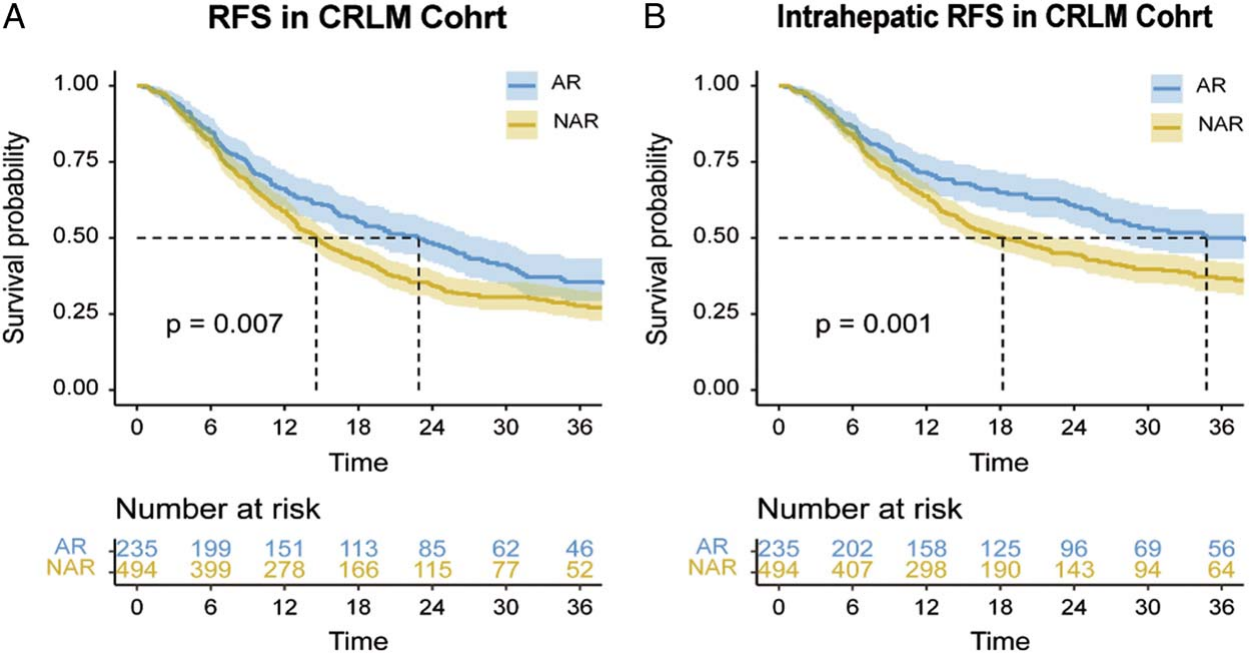
Kaplan–Meier estimates of RFS and intrahepatic RFS stratified by the type of resection in the total CRLM Cohort. (A) RFS curve for AR and NAR in CRLM Cohort; (B) Intrahepatic RFS curve for AR and NAR in CRLM Cohort. The shaded part of the survival curve represents a 95% confidence interval. AR, anatomical resection; CRLM, colorectal liver metastases; NAR, non-anatomical resection; RFS, relapse-free survival.

The median duration of follow-up was 33.1 months in the total CRLM Cohort. The 1-year, 2-year, and 3-year RFS were 61.2%, 39.2%, and 30.2%, respectively; the 1-year, 2-year, and 3-year intrahepatic RFS were 66.3%, 50.0%, and 41.1%, respectively. The median RFS for patients undergoing AR and NAR was 22.9 and 14.6 months, respectively (HR=0.762, 95% CI 0.625–0.928; *P*=0.007) (Fig. 1A, Supplementary Table S8, Supplemental Digital Content 2, http://links.lww.com/JS9/A830). The median intrahepatic RFS for patients undergoing AR and NAR was 34.8 and 18.2 months, respectively (HR=0.679, 95% CI 0.543–0.849; *P*=0.001) (Fig. 1B, Supplementary Table S9, Supplemental Digital Content 2, http://links.lww.com/JS9/A830). Significant differences of RFS and intrahepatic RFS between AR and NAR groups in the CRLM Cohort should be interpreted with caution because of the imbalance in the CRLM characteristics between AR and NAR groups (larger number of CRLM, more bilateral CRLM, and more gene mutation in NAR group).

### Subgroup analyses of RFS and intrahepatic RFS between AR and NAR groups

**Figure 2:**
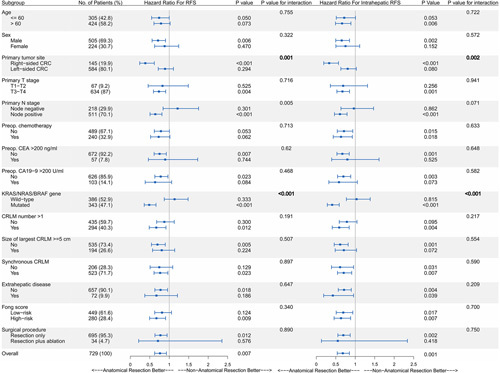
Hazard ratios for RFS (A) and intrahepatic RFS (B) in predefined subgroups of CRLM Cohort. For each subgroup in the forest plots, the square represents the point estimate of the treatment effect, and the horizontal line represents the 95% confidence interval. CRC, colorectal cancer; CRLM, colorectal liver metastases; RFS, relapse-free survival.

Results for RFS and intrahepatic RFS in predefined subgroups of the CRLM Cohort were generally consistent with those in the total CRLM Cohort (Fig. 2). The patients with gene (*KRAS/NRAS/BRAF*) mutation or right-sided colon cancer who underwent AR had a markedly improved RFS (gene mutation: median survival, 23.2 vs. 11.1 months, *P*<0.001; right-sidedness: median survival, 31.6 vs. 11.5 months, *P*<0.001) and intrahepatic RFS (gene mutation: median survival, >36 vs. 13.4 months, *P*<0.001; right-sidedness: median survival, >36 vs. 11.7 months, *P*<0.001). In the primary N-stage group, the test for interaction in RFS was significant (*P* value for interaction, 0.005), but weak evidence was shown in intrahepatic RFS subgroup analysis (borderline significance; *P* value for interaction, 0.071). Different from the primary N stage, *KRAS/NRAS/BRAF* gene group and primary tumor site group showed evidence of heterogeneity, with significant tests for interaction in RFS and intrahepatic RFS (*P* values for interaction were close to or less than 0.001). Therefore, the subgroups of these two variables would be further analyzed.

### Efficacy results of AR in gene-mutated subgroups

**Figure 3:**
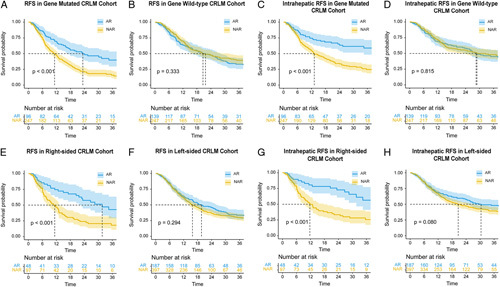
Kaplan–Meier estimates of RFS and intrahepatic RFS stratified by the type of resection in subgroups of CRLM Cohorts. (A, C) RFS and intrahepatic RFS in patients with gene (*KRAS/NRAS/BRAF*) mutated tumors grouped according to a type of resection in CRLM Cohort; (B, D) RFS and intrahepatic RFS in patients with gene wild-type tumors grouped according to a type of resection in CRLM Cohort; (E, G) RFS and intrahepatic RFS in patients with right-sided CRC grouped according to type of resection in CRLM Cohort; (F, H) RFS and intrahepatic RFS in patients with left-sided CRC grouped according to a type of resection in CRLM Cohort. AR, anatomical resection; CRLM, colorectal liver metastases; NAR, non-anatomical resection; RFS, relapse-free survival.

**Figure 4:**
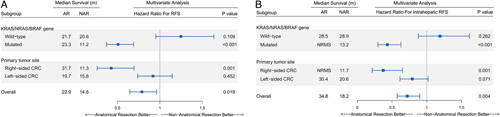
Adjusted hazard ratios after multivariate analysis for RFS (A) and intrahepatic RFS (B) in predefined subgroups of CRLM Cohort. For each subgroup in the forest plots, the square represents the point estimate of the treatment effect, and the horizontal line represents the 95% confidence interval. AR, anatomical resection; CRC, colorectal cancer; CRLM, colorectal liver metastases; NAR, non-anatomical resection; NRMS, not reach median survival; RFS, relapse-free survival.

Univariable and multivariable analyses were conducted in the gene-mutated (RFS, Supplementary Table S10, Supplemental Digital Content 2, http://links.lww.com/JS9/A830; intrahepatic RFS, Supplementary Table S11, Supplemental Digital Content 2, http://links.lww.com/JS9/A830), and gene wild-type subgroups cohorts of CRLM Cohorts (RFS, Supplementary Table S12, Supplemental Digital Content 2, http://links.lww.com/JS9/A830; intrahepatic RFS, Supplementary Table S13, Supplemental Digital Content 2, http://links.lww.com/JS9/A830). In the gene-mutated CRLM Cohort, patients who underwent AR had markedly improved RFS (HR: 0.490; 95% CI, 0.360–0.667; *P*<0.001) and intrahepatic RFS (HR: 0.410; 95% CI, 0.283–0.584; *P*<0.001) compared with patients who underwent NAR (Fig. 3A, C). Upon the multivariable analysis, the performance of AR remained prognostic independently for superior RFS and intrahepatic RFS (Fig. 4). In contrast, in the subgroup cohorts of patients with gene wild-type tumors, patients who underwent AR had similar RFS and intrahepatic RFS compared with those who underwent NAR (*P*>0.05, no significant differences) (Figs 3B, D and 4).

### Efficacy results of AR in right-sided CRC subgroups

Univariable and multivariable analyses were conducted in the right-sided (RFS, Supplementary Table S14, Supplemental Digital Content 2, http://links.lww.com/JS9/A830; intrahepatic RFS, Supplementary Table S15, Supplemental Digital Content 2, http://links.lww.com/JS9/A830) and left-sided (RFS, Supplementary Table S16, Supplemental Digital Content 2, http://links.lww.com/JS9/A830; intrahepatic RFS, Supplementary Table S17, Supplemental Digital Content 2, http://links.lww.com/JS9/A830) CRC subgroups cohorts of CRLM Cohort. In the right-sided CRC CRLM Cohort, patients who underwent AR had markedly improved RFS (HR: 0.403; 95% CI, 0.250–0.648; *P*<0.001) and intrahepatic RFS (HR: 0.340; 95% CI, 0.196–0.589; *P*<0.001) compared with patients who underwent NAR (Fig. 3E, G). In contrast, in the subgroup cohorts of patients with left-sided tumors, patients who underwent AR had similar RFS and intrahepatic RFS compared with those who underwent NAR (*P*>0.05, no significant differences) (Figs 3F, H and 4).

## Discussion

In our current study, we evaluated the role of AR among patients with CRLM, based on the preplanned subgroup analyses. Then we, for the first time, found that the performance of NAR was strongly associated with worse RFS and a higher intrahepatic relapse rate in the subgroup cohorts of patients with gene mutation and right-sided colon cancer. In contrast, patients with one of the two prognosis factors could be advised to undergo AR. Our research may help settle the issue regarding the selection of AR or NAR.

The extent of hepatectomy for CRLM has been a long-debated topic and an unsolved issue because previous studies have shown contradictory findings on the benefits or deficiencies of either approach^7,8,21^. Previous studies mostly focused on the technical aspects of the extent of hepatectomy rather than the biology of the tumor itself^7,8,21^. Margonis *et al*.^12^ analyzed the outcomes of AR and NAR based on the biologic marker (*KRAS* mutations). Because ‘colorectal tumor cells have been hypothesized to utilize the portal venous network during their metastatic spread^22,23^, Margonis *et al*.^12^ believed, ‘Intrahepatic metastasis via vascular dissemination is considered a key prognostic determinant among patients with CRLM’^24,25^. Margonis *et al*. stated, ‘the systematic removal of potentially “tumor-bearing” portal tributaries, through the performance of an AR would theoretically be expected to limit the development of intrahepatic recurrence or metastasis^12–15^. Due to their aggressive biological nature, *KRAS*-mutated CRLM were prone to vascular invasion and hematogenous metastasis^26^. Likewise, Margonis *et al*.^12^ proved that the performance of AR would manage *KRAS*-mutated CRLM more successfully.

In the cohort of patients with gene-mutated tumors, our investigation indicated the potential oncologic advantages of AR. In the study reported by Margonis *et al*.^12^, only one biologic variable, *KRAS* mutational status, was tested. As is well known, *KRAS*, *NRAS*, and *BRAF* are kinases on the RAS–RAF–MAPK signaling pathway. If *KRAS/NRAS/BRAF* genes are mutated, the downstream MAPK pathway will be activated continually, leading to tumor cell proliferation and development^27^. *KRAS/NRAS/BRAF* genes were considered important biomarkers that determined tumor biology and could be used to predict outcomes following CRLM resection^28^. Hence, not only *KRAS* but also *NRAS* and *BRAF* were incorporated into our analysis. As anticipated, Margonis’ findings were explored and verified. However, Joechle *et al*.^29^. considered that AR was not required for CRLM with RAS mutation in the analysis. Due to the early age of these patients, the method of RAS mutation testing in this study may have differed from ours, which would have affected the categorization of the study population. Furthermore, the AR in this study were defined imprecisely as ‘any formal segmentectomy and sectionectomy’, giving results that were contradictory to our own.

What has been more interesting is that for patients with right-sided CRC, AR is warranted due to the aggressive nature of right-sidedness. The location of the primary tumor might serve as a proxy for tumor molecular biology^30^. According to genetic and molecular analysis, CRC is no longer regarded as a single entity. The different pathways have been illustrated by The Cancer Genome Atlas in the distribution of the consensus molecular subtypes between midgut origin and hindgut origin CRCs^31^. Multiple biological variables have been proposed for the worse prognosis of patients with right-sidedness, including high frequencies of *TGFbR2* mutations, CpG island methylation, and *ERCC1* expression, which were all regarded as indicators of aggressive tumor biological behavior^32–35^. The location of the primary tumor is also an important prognostic factor in CRLM. In a meta-analysis of 66 studies including 1.43 million patients with all stages of CRC, right-sided CRC was associated with a significantly increased risk of death^36^. The embryonic midgut origin CRC is associated with a worse prognosis after hepatectomy^33,37–40^. This effect was even deemed independent of the RAS mutation status^40^. As a result, the role of AR was analyzed in the right-sided and left-sided CRC subgroup cohorts. Then we found that the performance of AR could control the aggressive nature of right-sidedness in CRLM.

The choice between AR or NAR depends on many factors, such as the number of nodules and their locations. For example, a surgeon may tend to choose to perform NAR on a patient with multiple or bilateral liver metastases and AR on a patient with a disease location in the left lobe. Since the number of liver metastases was regarded as a strong confounder in this study, great care was taken to control for the CRLM number (ranging from 1 to 3). It has to be acknowledged that the oncologic benefit of AR in this study is limited to patients with a small number of liver metastases (less than three). Yet, we think that our study’s findings can serve as a guide for surgeons in cases where a patient has the opportunity to perform either AR or NAR surgery.

Of course, there are certain limitations to this study. Firstly, this is a retrospective study and selection bias might exist in the current study. Secondly, the analyses should be cautiously considered owing to the limited enrollment number. The sample size of right-sided CRC was not large enough. Thirdly, data about resection margin width and margin status was incomplete and the relationship between resection margin width and aggressive biological behavior of tumors needed to be explored.

## Conclusion

Our findings support the premise that patients with gene mutations or right-sidedness may benefit more from the performance of AR, which may be useful when the operative plan is determined (seen in Supplementary Fig. S2, Supplemental Digital Content 2, http://links.lww.com/JS9/A830). To this end, additional studies are needed to confirm the findings, and a randomized controlled trial will be designed and initiated in our institution.

## Ethical approval

Ethical approval for this study was provided by the Ethical Committee of Zhongshan Hospital Fudan University, Shanghai, China, on 20 December 2015 (B2015-124R2).

## Sources of funding

Supported by The National Natural Science Foundation of China (82072653, 81602035, 82072678); Shanghai Science and Technology Committee Project (19511121300, 17411951300); Clinical Research Plan of SHDC (No. SHDC2020CR5006); Shanghai Engineering Research Center of Colorectal Cancer Minimally Invasive (17DZ2252600); The National Key R&D Program of China (2017YFC0908200).

## Author contribution

W.C., J.F., Y.W., and J.X.: concept and design; Y.C., S.Z., L.R., Y.X., D.Z., W.T., Q.Y., X.W., and J.X.: acquisition, analysis, or interpretation of data; W.C., Y.C., S.Z., L.R., and Y.X.: drafting of the manuscript; W.C., Y.W., and J.X.: critical revision of the manuscript for important intellectual content; Y.C. and W.C.: statistical analysis; L.R., Q.Y., X.W., J.F., and Y.W.: administrative, technical, or material support; J.X.: supervision.

## Conflicts of interest disclosure

The authors declare that they have no conflicts of interest.

## Research registration unique identifying number (UIN)

The study has registered with ClinicalTrials.gov, study number NCT05673564. Anatomical Resection in Colorectal Liver Metastases Patients - Full Text View - ClinicalTrials.gov.

## Guarantor

Dr Jianmin Xu had full access to all of the data in the study and takes responsibility for the integrity of the data and the accuracy of the data analysis.

## Provenance and peer review

Not commissioned, externally peer-reviewed.

## Data availability statement

The datasets used and analyzed during the current study are available from the corresponding author upon reasonable request.

## Supplementary Material

SUPPLEMENTARY MATERIAL
