# Supplementary material for: Anatomical resection improves relapse-free survival in colorectal liver metastases in patients with KRAS/NRAS/BRAF mutations or right-sided colon cancer: a retrospective cohort study
Source: Int J Surg. 2023 Aug 1;109(10):3070–7. doi: 10.1097/JS9.0000000000000562 (PMC10583959; doi:10.1097/JS9.0000000000000562)
Supplement: SUPPLEMENTARY MATERIAL [file js9-109-3070-s002.pdf]

A list of supplementary materials:

Figure S1. Patient selection. 1

Figure S2. Graphical abstract 2

Table S1. Summary of KRAS, NRAS, and BRAF mutations tested by ARMS 3

Table S2. Frequency of Exon Mutations in KRAS, NRAS, and BRAF 3

Table S3. Quality of Liver Resection, Short-Term Outcomes, and Chemotherapy 4

Table S4. Baseline characteristics in the gene mutated CRLM Cohort 5

Table S5. Baseline characteristics in the gene wild-type CRLM Cohort 6

Table S6. Baseline characteristics in the right-sided CRC CRLM Cohort 8

Table S7. Baseline characteristics in the left-sided CRC CRLM Cohort 8

Table S8. Uni- and Multivariable Predictors of RFS in the CRLM Cohort 9

Table S9. Uni- and Multivariable Predictors of Intrahepatic RFS in the CRLM Cohort 10

Table S10. Uni- and Multivariable Predictors of RFS in the gene mutated CRLM Cohort 10

Table S11. Uni- and Multivariable Predictors of Intrahepatic RFS in the gene mutated CRLM Cohort 12

Table S12. Uni- and Multivariable Predictors of RFS in the gene wild-type CRLM Cohort 13

Table S13. Uni- and Multivariable Predictors of Intrahepatic RFS in the gene wild-type CRLM Cohort 12

Table S14. Uni- and Multivariable Predictors of RFS in the Right-sided CRC CRLM Cohort 13

Table S15. Uni- and Multivariable Predictors of Intrahepatic RFS in the Right-sided CRC CRLM Cohort 13

Table S16. Uni- and Multivariable Predictors of RFS in the Left-sided CRC CRLM Cohort 14

Table S17. Uni- and Multivariable Predictors of Intrahepatic RFS in the Left-sided CRC CRLM Cohort 15

Figure S1. Patient selection. AR, anatomical resection; NAR, non-anatomic resection; Mutation, KRAS/NRAS/BRAF mutated tumors; Wild, KRAS/NRAS/BRAF wild-type tumors; Right-sided, right-sided CRC; Left-sided, left-sided CRC.

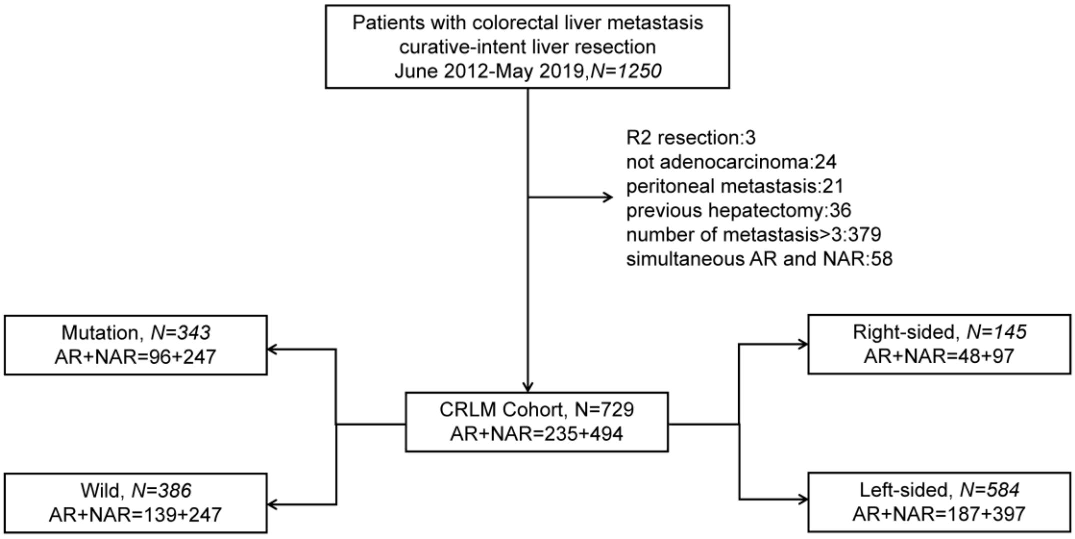

Figure S2. Graphical abstract

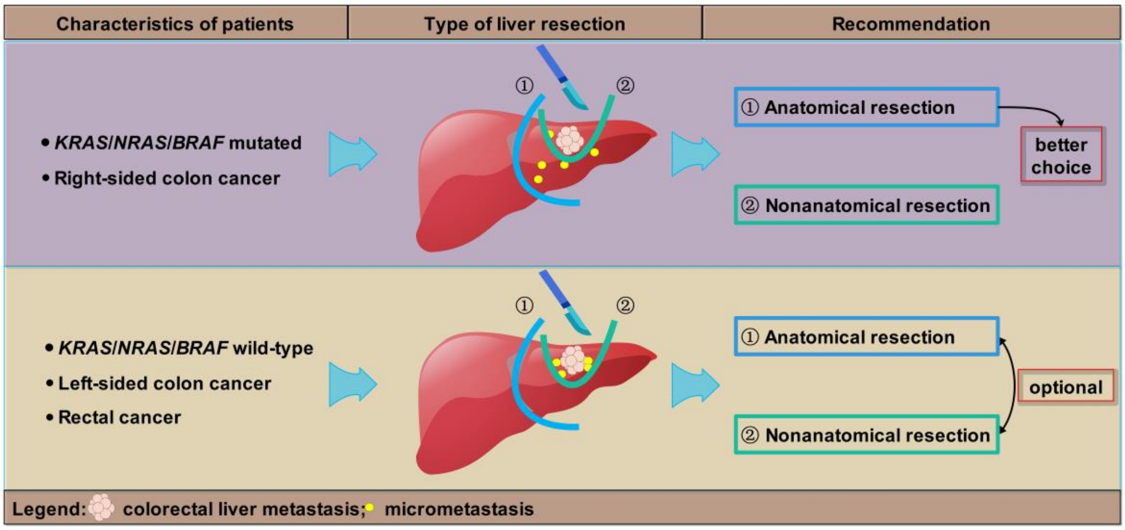

Table S1. Summary of KRAS, NRAS, and BRAF mutations tested by ARMS

Gene	Exon	Mutation loci
KRAS	2	G12S, G12D, G12C, G12R, G12V, G12A
	2	G13C, G13D
	3	Q61L, Q61R, Q61H
	4	K117N, A146T, A146V, A146P
NRAS	2	G12S, G12D
	2	G13D
	2	G12A, G12V, G12C, G13R, G13V
	3	Q61K, Q61H, Q61L, Q61R
	4	A146T
BRAF	15	V600E

The Amplification Refractory Mutation System (ARMS) technology is a PCR-based method that is used to detect specific mutations or genetic variations in a DNA sample. The ARMS technology works by designing primers that are specific to the mutated or variant allele of interest. When these primers are used in a PCR reaction, they amplify only the DNA fragment containing the mutation or variant, while not amplifying the wild-type or normal allele.

The ARMS technology typically involves the following steps:

1. Primer design: Two sets of primers are designed for each variant of interest. One set of primers is specific to the wild-type or normal allele, and the other set is specific to the mutant or variant allele. The primers are designed in such a way that they anneal to the DNA fragment adjacent to the site of the mutation or variation.

2. PCR amplification: The PCR reaction is carried out using the two sets of primers. The reaction conditions are optimized to ensure that only the specific allele is amplified. If the sample contains the wild-type allele, only the primers specific to the wild-type allele will amplify the target DNA fragment, while the primers specific to the mutant allele will not amplify any DNA fragment. Similarly, if the sample contains the mutant allele, only the primers specific to the mutant allele will amplify the target DNA fragment, while the primers specific to the wild-type allele will not amplify any DNA fragment.

3. Gel electrophoresis: The PCR products are then separated on an agarose gel using gel electrophoresis. The gel is stained with a DNA-specific dye to visualize the amplified DNA fragments. If the sample contains the wild-type allele, a single band of the expected size will be observed corresponding to the PCR product amplified by the wild-type allele-specific primers. If the sample contains the mutant allele, a single band of the expected size will be observed corresponding to the PCR product amplified by the mutant allele-specific primers.

Table S2. Frequency of Exon Mutations in *KRAS*, *NRAS*, and *BRAF*

Exon Mutations	Total (*N*=729)	AR (*N* =235)	NAR (*N* =494)
KRAS mutated	306 (42.0)	88 (37.4)	218 (44.1)
Exon 2 mutation	265 (36.4)	80 (34.0)	185 (37.4)
Exon 3 mutation	19 (2.6)	4 (1.7)	15 (3.0)
Exon 4 mutation	22 (3.0)	4 (1.7)	18 (3.6)
NRAS mutated	23 (3.2)	4 (1.7%)	19 (3.8)
Exon 2 mutation	10 (1.4)	2 (0.9)	8 (1.6)
Exon 3 mutation	12 (1.6)	2 (0.9)	10 (2.0)
Exon 4 mutation	1 (0.1)	0	1 (0.2)
BRAF mutated	17 (2.3)	4 (1.7)	13 (2.6)
Exon 15: V600E	17 (2.3)	4 (1.7)	13 (2.6)

Table S3. Quality of Liver Resection, Short-Term Outcomes, and Chemotherapy

	Total (*N*=729)	AR (*N* =235)	NAR (*N* =494)
R0 resection for Liver, n (%)	729 (100.0)	235 (100.0)	494 (100.0)
Estimated blood loss> 300 ml, n (%)	86 (11.8)	25 (10.6)	61 (12.3)
Blood transfusion, n (%)	6 (0.8)	2 (0.9)	4 (0.8)
Major complications, n (%)	81 (11.1)	26 (11.1)	55 (11.1)
Mortality, n (%)	0 (0.0)	0 (0.0)	0 (0.0)
Cycles of chemotherapy, n (%)			
0	5 (0.7)	2 (0.9)	3 (0.6)
1-6	48 (6.6)	15 (6.4)	33 (6.7)
7-12	676 (92.7)	218 (92.8)	458 (92.7)
Type of chemotherapy, n (%)			
Only Capecitabine	23 (3.2)	9 (3.8)	14 (2.8)
Capox	69 (9.5)	24 (10.2)	45 (9.1)
FOLFOX	339 (46.5)	110 (46.8)	229 (46.3)
FOLFIRI	45 (6.2)	14 (6.0)	31 (6.3)
Doublet Chemo. + target agents	236 (32.4)	71 (30.2)	165 (33.4)
Other	12 (1.6)	5 (2.1)	7 (1.4)

Table S4. Baseline characteristics in the gene mutated CRLM Cohort

	Total (*N*=343)	AR (*N* =96)	NAR (*N* =247)	*P*
Patient characteristics, n (%)				
Age > 60 years	204 (59.5)	59 (61.5)	145 (58.7)	0.641
Female	117 (34.1)	37 (38.5)	80 (32.4)	0.281
Primary tumor characteristics, n (%)				
Right-sided CRC	99 (28.9)	30 (31.3)	69 (27.9)	0.543
Left-sided CRC	244 (71.1)	66 (68.8)	178 (72.1)	
T stage: T1-T2	31 (9.0)	11 (11.5)	20 (8.1)	
T stage: T3-T4	297 (86.6)	84 (87.5)	213 (86.2)	0.400
T stage: unknown	15 (4.4)	1 (1.0)	14 (5.7)	
N stage: node negative	105 (30.6)	36 (37.5)	69 (27.9)	0.084
N stage: node positive	238 (69.4)	60 (62.5)	178 (72.1)	
Preoperative factors, n (%)				
Preop. chemotherapy	105 (30.6)	34 (35.4)	71 (28.7)	0.229
Preop. CEA >200 ng/ml	26 (7.6)	9 (9.4)	17 (6.9)	0.434
Preop. CA19-9 >200 U/ml	63 (18.4)	25 (26.0)	38 (15.4)	0.022
KRAS/NRAS/BRAF mutated, n (%)				
KRAS mutated	305 (88.9)	88 (91.7)	217 (87.9)	0.313
NRAS mutated	23 (6.7)	4 (4.2)	19 (7.7)	0.241
BRAF mutated	17 (5.0)	4 (4.2)	13 (5.3)	0.886
CRLM characteristics, n (%)				
Number of CRLM				
1	202 (58.9)	65 (67.7)	137 (55.5)	
2	89 (25.9)	21 (21.9)	68 (27.5)	0.101
3	52 (15.2)	10 (10.4)	42 (17.0)	
Size of largest CRLM≥5cm	69 (20.1)	29 (30.2)	40 (16.2)	0.004
Bilateral CRLM[a]	72 (21.0)	9 (9.4)	63 (25.5)	0.001
Synchronous CRLM	240 (70.0）	69 (71.9)	171 (69.2)	0.631
Extrahepatic disease, n (%)	40 (11.7)	16 (16.7)	24 (9.7)	0.072
Fong score, n (%)				
Low-risk	105 (30.6)	32 (33.3)	73 (29.6)	
Medium-risk	209 (60.9)	54 (56.3)	155 (62.8)	0.494
High-risk	29 (8.5)	10 (10.4)	19 (7.7)	
Surgical procedure, n (%)				

Resection only	328 (95.6)	94 (97.9)	234 (94.7)	0.318
Resection plus ablation	15 (4.4)	2 (2.1)	13 (5.3)	

a. If the CRLM is located on the boundary line between the left lobe and the right lobe of liver, it is considered as bilateral metastasis; Abbreviations: AR, Anatomical Resection; NAR, Non-Anatomical Resection; CEA, Carcinoembryonic Antigen; CRLM, colorectal liver metastasis.

Table S5. Baseline characteristics in the gene wild-type CRLM Cohort

	Total (*N*=386)	AR (*N* =139)	NAR (*N* =247)	*P*
Patient characteristics, n (%)				
Age > 60 years	220 (57.0)	84 (60.4)	136 (55.1)	0.306
Female	107 (27.7)	38 (27.3)	69 (27.9)	0.900
Primary tumor characteristics, n (%)				
Right-sided CRC	46 (11.9)	18 (12.9)	28 (11.3)	0.639
Left-sided CRC	340 (88.1)	121 (87.1)	219 (88.7)	
T stage: T1-T2	36 (9.3)	15 (10.8)	21 (8.5)	
T stage: T3-T4	337 (87.3)	121 (87.1)	216 (87.4)	0.495
T stage: unknown	13 (3.4)	3 (2.2)	10 (4.0)	
N stage: node negative	113 (29.3)	43 (30.9)	70 (28.3)	0.591
N stage: node positive	273 (70.7)	96 (69.1)	177 (71.7)	
Preoperative factors, n (%)				
Preop. chemotherapy	135 (35.0)	40 (28.8)	95 (38.5)	0.055
Preop. CEA >200 ng/ml	31 (8.0)	10 (7.2)	21 (8.5)	0.650
Preop. CA19-9 >200 U/ml	40 (10.4)	12 (8.6)	28 (11.3)	0.403
KRAS/NRAS/BRAF mutated, n (%)	0 (0.0)	0 (0.0)	0 (0.0)	-
KRAS mutated	0 (0.0)	0 (0.0)	0 (0.0)	-
NRAS mutated	0 (0.0)	0 (0.0)	0 (0.0)	-
BRAF mutated	0 (0.0)	0 (0.0)	0 (0.0)	-
CRLM characteristics, n (%)				
Number of CRLM				
1	233 (60.4)	94 (67.6)	139 (56.3)	
2	102 (26.4)	36 (25.9)	66 (26.7)	0.009
3	51 (13.2)	9 (6.5)	42 (17.0)	
Size of largest CRLM⩾5cm	125 (32.4)	69 (49.6)	56 (22.7)	0.000
Bilateral CRLM[a]	89 (23.1)	22 (15.8)	67 (27.1)	0.011

Synchronous CRLM	283 (73.3)	104 (74.8)	179 (72.5)	0.616
Extrahepatic disease, n (%)	32 (8.3)	6 (4.3)	26 (10.5)	0.034
Fong score, n (%)				
Low-risk	94 (24.4)	34 (24.5)	60 (24.3)	
Medium-risk	251 (65.0)	85 (61.2)	166 (67.2)	0.182
High-risk	41 (10.6)	20 (14.4)	21 (8.5)	
Surgical procedure, n (%)				
Resection only	367 (95.1)	137 (98.6)	230 (93.1)	0.018
Resection plus ablation	19 (4.9)	2 (1.4)	17 (6.9)	

a. If the CRLM is located on the boundary line between the left lobe and the right lobe of liver, it is considered as bilateral metastasis; Abbreviations: AR, Anatomical Resection; NAR, Non-Anatomical Resection; CEA, Carcinoembryonic Antigen; CRLM, colorectal liver metastasis.

Table S6. Baseline characteristics in the right-sided CRC CRLM Cohort

	Total (*N*=145)	AR (*N* =48)	NAR (*N* =97)	*P*
Patient characteristics, n (%)				
Age > 60 years	93 (64.1)	33 (68.8)	60 (61.9)	0.415
Female	52 (35.9)	17 (35.4)	35 (36.1)	0.937
Primary tumor characteristics, n (%)				
Right-sided CRC	145 (100.0)	48 (100.0)	97 (100.0)	-
Left-sided CRC	0 (0.0)	0 (0.0)	0 (0.0)	
T stage: T1-T2	5 (3.4)	3 (6.3)	2 (2.1)	
T stage: T3-T4	134 (92.4)	45 (93.8)	89 (91.8)	0.459
T stage: unknown	6 (4.1)	0 (0.0)	6 (6.2)	
N stage: node negative	59 (40.7)	26 (54.2)	33 (34.0)	0.020
N stage: node positive	86 (59.3)	22 (45.8)	64 (66.0)	
Preoperative factors, n (%)				
Preop. chemotherapy	39 (26.9)	13 (27.1)	26 (26.8)	0.972
Preop. CEA >200 ng/ml	9 (6.2)	3 (6.3)	6 (6.2)	1.000
Preop. CA19-9 >200 U/ml	26 (17.9)	9 (18.8)	17 (17.5)	0.856
KRAS/NRAS/BRAF mutated, n (%)	99 (68.3)	30 (62.5)	69 (71.1)	0.293
KRAS mutated	86 (59.3)	27 (56.3)	59 (60.8)	0.598
NRAS mutated	4 (2.8)	1 (2.1)	3 (3.1)	1.000
BRAF mutated	10 (6.9)	2 (4.2)	8 (8.2)	0.573

CRLM characteristics, n (%)				
Number of CRLM				
1	97 (66.9)	33 (68.8)	64 (66.0)	
2	29 (20.0)	11 (22.9)	18 (18.6)	0.452
3	19 (13.1)	4 (8.3)	15 (15.5)	
Size of largest CRLM⩾5cm	43 (29.7)	23 (47.9)	20 (20.6)	0.001
Bilateral CRLM[a]	24 (16.6)	5 (10.4)	19 (19.6)	0.162
Synchronous CRLM	108 (74.5)	33 (68.8)	75 (77.3)	0.265
Extrahepatic disease, n (%)	13 (9.0)	6 (12.5)	7 (7.2)	0.460
Fong score, n (%)				
Low-risk	46 (31.7)	19 (39.6)	27 (27.8)	
Medium-risk	88 (60.8)	24 (50.0)	64 (66.0)	0.177
High-risk	11 (7.6)	5 (10.4)	6 (6.2)	
Surgical procedure, n (%)				
Resection only	137 (94.5)	48 (100.0)	89 (91.8)	0.097
Resection plus ablation	8 (5.5)	0 (0.0)	8 (8.2)	

a. If the CRLM is located on the boundary line between the left lobe and the right lobe of liver, it is considered as bilateral metastasis; Abbreviations: AR, Anatomical Resection; NAR, Non-Anatomical Resection; CEA, Carcinoembryonic Antigen; CRLM, colorectal liver metastasis.

Table S7. Baseline characteristics in the left-sided CRC CRLM Cohort

	Total (*N*=584)	AR (*N* =187)	NAR (*N* =397)	*P*
Patient characteristics, n (%)				
Age > 60 years	331 (56.7)	110 (58.8)	221 (55.7)	0.473
Female	172 (29.5)	58 (31.0)	114 (28.7)	0.569
Primary tumor characteristics, n (%)				
Right-sided CRC	0 (0.0)	0 (0.0)	0 (0.0)	-
Left-sided CRC	584 (100.0)	187 (100.0)	397 (100.0)	
T stage: T1-T2	62 (10.6)	23 (12.3)	39 (9.8)	
T stage: T3-T4	500 (85.6)	160 (85.6)	340 (85.6)	0.419
T stage: unknown	22 (3.8)	4 (2.1)	18 (4.5)	
N stage: node negative	159 (27.2)	53 (28.3)	106 (26.7)	0.677
N stage: node positive	425 (72.8)	134 (71.7)	291 (73.3)	
Preoperative factors, n (%)				

Preop. chemotherapy	201 (34.4)	61 (32.6)	140 (35.3)	0.530
Preop. CEA >200 ng/ml	48 (8.2)	16 (8.6)	32 (8.1)	0.839
Preop. CA19-9 >200 U/ml	77 (13.2)	28 (15.0)	49 (12.3)	0.381
KRAS/NRAS/BRAF mutated, n (%)	244 (41.8)	66 (35.3)	178 (44.8)	0.029
KRAS mutated	220 (37.7)	61 (32.6)	159 (40.1)	0.084
NRAS mutated	19 (3.3)	3 (1.6)	16 (4.0)	0.123
BRAF mutated	7 (1.2)	2 (1.1)	5 (1.3)	1.000
CRLM characteristics, n (%)				
Number of CRLM				
1	338 (57.9)	126 (67.4)	212 (53.4)	
2	162 (27.7)	46 (24.6)	116 (29.2)	0.001
3	84 (14.4)	15 (8.0)	69 (17.4)	
Size of largest CRLM⩾5cm	151 (25.9)	75 (40.1)	76 (19.1)	0.000
Bilateral CRLM[a]	137 (23.5)	26 (13.9)	111 (28.0)	0.000
Synchronous CRLM	415 (71.1)	140 (74.9)	275 (69.3)	0.164
Extrahepatic disease, n (%)	59 (10.1)	16 (8.6)	43 (10.8)	0.395
Fong score, n (%)				
Low-risk	153 (26.2)	47 (25.1)	106 (26.7)	
Medium-risk	372 (63.7)	115 (61.5)	257 (64.7)	0.199
High-risk	59 (10.1)	25 (13.4)	34 (8.6)	
Surgical procedure, n (%)				
Resection only	558 (95.5)	183 (97.9)	375 (94.5)	0.063
Resection plus ablation	26 (4.5)	4 (2.1)	22 (5.5)	

a. If the CRLM is located on the boundary line between the left lobe and the right lobe of liver, it is considered as bilateral metastasis; Abbreviations: AR, Anatomical Resection; NAR, Non-Anatomical Resection; CEA, Carcinoembryonic Antigen; CRLM, colorectal liver metastasis.

Table S8. Uni- and Multivariable Predictors of RFS in the CRLM Cohort

	Univariable			Multivariable		
	**HR**	**95% CI**	***P***	**HR**	**95% CI**	***P***
Age > 60 years	0.801	0.668-0.961	0.017	0.794	0.661-0.954	0.014
Female sex	1.041	0.857-1.264	0.687			
Primary T3 or T4	0.896	0.663-1.212	0.476			
Primary node positive	1.546	1.254-1.905	0.000	1.504	1.217-1.859	0.000

Primary location: right-sided	1.133	0.905-1.420	0.276			
Preop. CEA >200 ng/ml	1.444	1.047-1.990	0.025	1.515	1.217-1.859	0.014
Preop. CA19-9 >200 U/ml	1.314	1.019-1.694	0.035	1.111	0.851-1.449	0.440
KRAS/NRAS/BRAF mutated	1.559	1.299-1.869	0.000	1.562	1.298-1.880	0.000
Number of metastasis > 1	1.411	1.177-1.692	0.000	1.298	1.079-1.563	0.006
Size of largest CRLM≥5cm	1.118	0.913-1.369	0.281			
Synchronous CRLM	1.080	0.882-1.322	0.456			
Extrahepatic disease	1.531	1.160-2.020	0.003	1.378	1.041-1.826	0.025
Ablation	1.385	0.945-2.030	0.095	1.200	0.813-1.771	0.358
Anatomical resection	0.762	0.625-0.928	0.007	0.788	0.646-0.961	0.019

RFS, relapse-free survival; HR, hazard ratio; CI, confidence interval; *P*, P value

Table S9. Uni- and Multivariable Predictors of Intrahepatic RFS in the CRLM Cohort

	Univariable			Multivariable		
	**HR**	**95% CI**	***P***	**HR**	**95% CI**	***P***
Age > 60 years	0.820	0.672-1.002	0.052	0.831	0.679-1.016	0.071
Female sex	1.006	0.810-1.248	0.960			
Primary T3 or T4	0.817	0.591-1.130	0.223			
Primary node positive	1.572	1.245-1.984	0.000	1.542	1.219-1.950	0.000
Primary location: right-sided	1.158	0.906-1.479	0.240			
Preop. CEA >200 ng/ml	1.536	1.094-2.157	0.013	1.637	1.163-2.303	0.005
Preop. CA19-9 >200 U/ml	1.226	0.922-1.631	0.162			
KRAS/NRAS/BRAF mutated	1.411	1.155-1.722	0.001	1.410	1.154-1.723	0.001
Number of metastasis > 1	1.402	1.148-1.711	0.001	1.255	1.023-1.540	0.029
Size of largest CRLM≥5cm	1.161	0.930-1.450	0.187			
Synchronous CRLM	1.125	0.899-1.407	0.304			
Extrahepatic disease	1.087	0.783-1.508	0.618			
Ablation	1.451	0.967-2.176	0.072	1.275	0.843-1.928	0.251
Anatomical resection	0.679	0.543-0.849	0.001	0.719	0.574-0.900	0.004

RFS, relapse-free survival; HR, hazard ratio; CI, confidence interval; *P*, P value

Table S10. Uni- and Multivariable Predictors of RFS in the gene mutated CRLM Cohort

	Univariable			Multivariable		
	**HR**	**95% CI**	***P***	**HR**	**95% CI**	***P***
Age > 60 years	0.668	0.519-0.861	0.002	0.684	0.531-0.882	0.003
Female sex	1.044	0.802-1.359	0.747			
Primary T3 or T4	0.908	0.600-1.376	0.650			
Primary node positive	1.582	1.185-2.112	0.002	1.457	1.088-1.949	0.011
Primary location: right-sided	1.016	0.768-1.345	0.910			
Preop. CEA >200 ng/ml	1.363	0.852-2.182	0.196			
Preop. CA19-9 >200 U/ml	1.054	0.758-1.465	0.756			
KRAS/NRAS/BRAF mutated	-	-	-	-	-	-
Number of metastasis > 1	1.240	0.962-1.598	0.097	1.139	0.882-1.471	0.320
Size of largest CRLM≥5cm	1.185	0.872-1.611	0.277			
Synchronous CRLM	1.019	0.774-1.341	0.892			
Extrahepatic disease	1.262	0.869-1.831	0.221			
Ablation	1.054	0.590-1.885	0.858			
Anatomical resection	0.490	0.360-0.667	0.000	0.506	0.371-0.690	0.000

RFS, relapse-free survival; HR, hazard ratio; CI, confidence interval; *P*, P value

Table S11. Uni- and Multivariable Predictors of Intrahepatic RFS in the gene mutated CRLM Cohort

	Univariable			Multivariable		
	**HR**	**95% CI**	***P***	**HR**	**95% CI**	***P***
Age > 60 years	0.717	0.542-0.949	0.020	0.782	0.549-0.964	0.027
Female sex	1.061	0.793-1.421	0.689			
Primary T3 or T4	0.819	0.520-1.290	0.388			
Primary node positive	1.549	1.121-2.140	0.008	1.419	1.025-1.965	0.035
Primary location: right-sided	1.041	0.765-1.416	0.800			
Preop. CEA >200 ng/ml	1.418	0.861-2.336	0.170			
Preop. CA19-9 >200 U/ml	0.973	0.670-1.415	0.887			
KRAS/NRAS/BRAF mutated	-	-	-	-	-	-
Number of metastasis > 1	1.225	0.926-1.623	0.156			
Size of largest CRLM≥5cm	1.251	0.889-1.760	0.199			

Synchronous CRLM	1.006	0.741-1.364	0.972			
Extrahepatic disease	0.665	0.403-1.064	0.087	0.692	0.426-1.126	0.138
Ablation	1.062	0.562-2.007	0.854			
Anatomical resection	0.410	0.283-0.594	0.000	0.432	0.297-0.628	0.000

RFS, relapse-free survival; HR, hazard ratio; CI, confidence interval; *P*, P value

Table S12. Uni- and Multivariable Predictors of RFS in the gene wild-type CRLM Cohort

	Univariable			Multivariable		
	**HR**	**95% CI**	***P***	**HR**	**95% CI**	***P***
Age > 60 years	0.920	0.707-1.196	0.531			
Female sex	0.951	0.710-1.273	0.734			
Primary T3 or T4	0.897	0.577-1.393	0.628			
Primary node positive	1.541	1.137-2.088	0.005	1.480	1.083-2.022	0.014
Primary location: right-sided	1.016	0.680-1.519	0.938			
Preop. CEA >200 ng/ml	1.568	1.010-2.436	0.045	1.637	1.035-2.589	0.035
Preop. CA19-9 >200 U/ml	1.554	1.039-2.324	0.032	1.467	0.963-2.234	0.075
KRAS/NRAS/BRAF mutated	-	-	-			
Number of metastasis > 1	1.585	1.221-2.057	0.001	1.614	1.238-2.102	0.000
Size of largest CRLM≥5cm	1.211	0.920-1.594	0.172			
Synchronous CRLM	1.182	0.875-1.596	0.275			
Extrahepatic disease	1.733	1.143-2.628	0.010	1.485	0.965-2.285	0.072
Ablation	1.772	1.064-2.950	0.028	1.814	1.075-3.063	0.026
Anatomical resection	1.140	0.874-1.488	0.333	1.248	0.952-1.637	0.109

RFS, relapse-free survival; HR, hazard ratio; CI, confidence interval; *P*, P value

Table S13. Uni- and Multivariable Predictors of Intrahepatic RFS in the gene wild-type CRLM Cohort

	Univariable			Multivariable		
	**HR**	**95% CI**	***P***	**HR**	**95% CI**	***P***
Age > 60 years	0.906	0.681-1.206	0.499			
Female sex	0.890	0.643-1.231	0.481			

Primary T3 or T4	0.826	0.520-1.314	0.420			
Primary node positive	1.610	1.150-2.256	0.006	1.636	1.154-2.318	0.006
Primary location: right-sided	1.104	0.720-1.695	0.649			
Preop. CEA >200 ng/ml	1.683	1.058-2.678	0.028	1.788	1.104-2.895	0.018
Preop. CA19-9 >200 U/ml	1.530	0.980-2.389	0.061	1.479	0.928-2.359	0.100
KRAS/NRAS/BRAF mutated	-	-	-			
Number of metastasis > 1	1.582	1.191-2.102	0.002	1.517	1.135-2.028	0.005
Size of largest CRLM≥5cm	1.229	0.912-1.657	0.175			
Synchronous CRLM	1.293	0.928-1.802	0.129			
Extrahepatic disease	1.774	1.137-2.768	0.012	1.481	0.935-2.346	0.094
Ablation	1.918	1.131-3.252	0.016	2.173	1.249-3.782	0.006
Anatomical resection	1.036	0.773-1.387	0.815	1.190	0.878-1.611	0.262

RFS, relapse-free survival; HR, hazard ratio; CI, confidence interval; *P*, P value

Table S14. Uni- and Multivariable Predictors of RFS in the Right-sided CRC CRLM Cohort

	**Univariable**			**Multivariable**		
	**HR**	**95% CI**	***P***	**HR**	**95% CI**	***P***
Age > 60 years	0.701	0.462-1.062	0.094	0.666	0.435-1.021	0.062
Female sex	0.989	0.649-1.507	0.960			
Primary T3 or T4	1.199	0.379-3.795	0.757			
Primary node positive	1.627	1.066-2.485	0.024	1.461	0.941-2.270	0.091
Primary location: right-sided	-	-	-	-	-	-
Preop. CEA >200 ng/ml	2.030	0.931-4.425	0.075	2.897	1.286-6.525	0.010
Preop. CA19-9 >200 U/ml	1.435	0.865-2.382	0.162			
KRAS/NRAS/BRAF mutated	1.508	0.964-2.359	0.072	1.536	0.973-2.427	0.066
Number of metastasis > 1	1.056	0.684-1.629	0.806			
Size of largest CRLM≥5cm	0.861	0.552-1.343	0.508			
Synchronous CRLM	0.922	0.584-1.457	0.728			
Extrahepatic disease	1.842	0.980-3.462	0.058	2.077	1.095-3.940	0.025
Ablation	1.119	0.488-2.564	0.791			
Anatomical resection	0.403	0.250-0.648	0.000	0.426	0.261-0.695	0.001

RFS, relapse-free survival; HR, hazard ratio; CI, confidence interval; *P*, P value

Table S15. Uni- and Multivariable Predictors of Intrahepatic RFS in the Right-sided CRC CRLM Cohort

	Univariable			Multivariable		
	**HR**	**95% CI**	***P***	**HR**	**95% CI**	***P***
Age > 60 years	0.692	0.442-1.083	0.107			
Female sex	0.927	0.585-1.467	0.745			
Primary T3 or T4	0.941	0.296-2.993	0.919			
Primary node positive	2.015	1.249-3.251	0.004	1.739	1.072-2.819	0.025
Primary location: right-sided	-	-	-	-	-	-
Preop. CEA >200 ng/ml	1.873	0.806-4.353	0.145			
Preop. CA19-9 >200 U/ml	1.331	0.767-2.308	0.309			
KRAS/NRAS/BRAF mutated	1.307	0.811-2.108	0.272			
Number of metastasis > 1	1.044	0.651-1.676	0.858			
Size of largest CRLM≥5cm	0.860	0.529-1.399	0.543			
Synchronous CRLM	1.145	0.684-1.916	0.606			
Extrahepatic disease	0.888	0.409-1.931	0.765			
Ablation	1.063	0.429-2.630	0.895			
Anatomical resection	0.340	0.196-0.589	0.000	0.373	0.214-0.651	0.001

RFS, relapse-free survival; HR, hazard ratio; CI, confidence interval; *P*, P value

Table S16. Uni- and Multivariable Predictors of RFS in the Left-sided CRC CRLM Cohort

	Univariable			Multivariable		
	**HR**	**95% CI**	***P***	**HR**	**95% CI**	***P***
Age > 60 years	0.822	0.671-1.007	0.059	0.827	0.674-1.014	0.068
Female sex	1.044	0.838-1.301	0.703			
Primary T3 or T4	0.861	0.628-1.180	0.351			
Primary node positive	1.568	1.229-1.999	0.000	1.515	1.193-1.948	0.001
Primary location: right-sided	-	-	-			
Preop. CEA >200 ng/ml	1.369	0.962-1.949	0.081	1.473	1.032-2.100	0.033
Preop. CA19-9 >200 U/ml	1.277	0.950-1.716	0.106			
KRAS/NRAS/BRAF mutated	1.569	1.279-1.924	0.000	1.530	1.245-1.879	0.000
Number of metastasis > 1	1.523	1.243-1.865	0.000	1.431	1.163-1.761	0.001

Size of largest CRLM≥5cm	1.210	0.963-1.521	0.102			
Synchronous CRLM	1.117	0.891-1.400	0.336			
Extrahepatic disease	1.473	1.082-2.006	0.014	1.334	0.975-1.826	0.071
Ablation	1.445	0.938-2.224	0.095	1.186	0.764-1.840	0.447
Anatomical resection	0.890	0.716-1.106	0.294	0.919	0.738-1.145	0.452

RFS, relapse-free survival; HR, hazard ratio; CI, confidence interval; *P*, P value

Table S17. Uni- and Multivariable Predictors of Intrahepatic RFS in the Left-sided CRC CRLM Cohort

	Univariable			Multivariable		
	**HR**	**95% CI**	***P***	**HR**	**95% CI**	***P***
Age > 60 years	0.845	0.675-1.057	0.141			
Female sex	1.022	0.800-1.305	0.865			
Primary T3 or T4	0.790	0.562-1.110	0.174			
Primary node positive	1.499	1.146-1.959	0.003	1.472	1.125-1.927	0.005
Primary location: right-sided	-	-	-	-	-	-
Preop. CEA >200 ng/ml	1.493	1.029-2.165	0.035	1.363	0.922-2.014	0.120
Preop. CA19-9 >200 U/ml	1.175	0.840-1.645	0.347			
KRAS/NRAS/BRAF mutated	1.417	1.132-1.775	0.002	1.440	1.144-1.812	0.002
Number of metastasis > 1	1.523	1.218-1.904	0.000	1.390	1.105-1.748	0.005
Size of largest CRLM≥5cm	1.263	0.983-1.622	0.068	1.403	1.066-1.847	0.016
Synchronous CRLM	1.115	0.869-1.430	0.394			
Extrahepatic disease	1.141	0.795-1.638	0.474			
Ablation	1.593	1.012-2.509	0.044	1.418	0.890-2.258	0.141
Anatomical resection	0.803	0.628-1.027	0.080	0.791	0.614-1.020	0.071

RFS, relapse-free survival; HR, hazard ratio; CI, confidence interval; *P*, P value
